# Timescales of Growth Response of Microbial Mats to Environmental Change in an Ice-Covered Antarctic Lake

**DOI:** 10.3390/biology2010151

**Published:** 2013-03-25

**Authors:** Ian Hawes, Dawn Y. Sumner, Dale T. Andersen, Anne D. Jungblut, Tyler J. Mackey

**Affiliations:** 1 Gateway Antarctica, University of Canterbury, Private Bag 4800, Christchurch, New Zealand; 2 Department of Geology, University of California, Davis, CA 95616, USA; E-Mails: dysumner@ucdavis.edu (D.Y.S.); tjmackey@ucdavis.edu (T.J.M.); 3 Carl Sagan Center for the Study of Life in the Universe, SETI Institute, 189 Bernado Avenue, Suite 100, Mountain View, CA 94043, USA; E-Mail: dandersen@carlsagancenter.org; 4 Department of Life Sciences, The Natural History Museum, Cromwell Road, London, UK; E-Mail: a.jungblut@nhm.ac.uk

**Keywords:** cyanobacteria, benthic communities, microbial mat, environmental change, Antarctic lake

## Abstract

Lake Vanda is a perennially ice-covered, closed-basin lake in the McMurdo Dry Valleys, Antarctica. Laminated photosynthetic microbial mats cover the floor of the lake from below the ice cover to >40 m depth. In recent decades, the water level of Lake Vanda has been rising, creating a “natural experiment” on development of mat communities on newly flooded substrates and the response of deeper mats to declining irradiance. Mats in recently flooded depths accumulate one lamina (~0.3 mm) per year and accrue ~0.18 µg chlorophyll-a cm^−2^ y^−1^. As they increase in thickness, vertical zonation becomes evident, with the upper 2-4 laminae forming an orange-brown zone, rich in myxoxanthophyll and dominated by intertwined *Leptolyngbya* trichomes. Below this, up to six phycobilin-rich green/pink-pigmented laminae form a subsurface zone, inhabited by *Leptolyngbya, Oscillatoria* and *Phormidium* morphotypes. Laminae continued to increase in thickness for several years after burial, and PAM fluorometry indicated photosynthetic potential in all pigmented laminae. At depths that have been submerged for >40 years, mats showed similar internal zonation and formed complex pinnacle structures that were only beginning to appear in shallower mats. Chlorophyll-a did not change over time and these mats appear to represent resource-limited “climax” communities. Acclimation of microbial mats to changing environmental conditions is a slow process, and our data show how legacy effects of past change persist into the modern community structure.

## 1. Introduction

The perennially ice covered lakes of the McMurdo Dry Valleys (MDVs), Antarctica, are amongst the most extreme lacustrine environments on Earth. Already set apart from most lakes by their endorheic and meromictic characters, the lakes also have extreme photoperiods, are persistently cold and are almost isolated from all physical forcing by perennial ice cover. In addition, they contain truncated, microbially-dominated food webs within which higher metazoans are largely absent. These conditions combine to result in minimal physical and biological perturbations of lake sediments, a situation that favors the formation of well-developed, complex microbial mat communities [1,2]. In response, microbial mats are both widespread and abundant in the lakes of the MDV [3,4], where they play a major role in carbon and nutrient cycling on a lake-wide basis [5,6,7]. 

While it is acknowledged that benthic microbial communities are significant components of the MDV lake systems, to date, quantitative information on their self-organization and growth is sparse, in part because accumulation rates tend to be slow and long duration observations would be required to assess how communities change. However, in recent decades, the MDV lakes have been undergoing gradual increases in lake level, in response to persistently warm summer temperatures [8,9]. This rising level provides a natural experiment into the rate and trajectory of microbial mat development on recently inundated soils and on the effects of changing environment on established microbial mat communities as levels increase. In Lake Joyce, for example, we have shown that reduced irradiant flux to deep mat communities has resulted in the extinction of distinctive components of the benthic biota, while inundation of substrates provide opportunities for colonization, albeit at a slow pace [10]. The relationship between the speed at which lake levels rise and how various communities respond has emerged as an important consideration in understanding ecosystem responses to change [7]. 

Lake Vanda, one of the MDV lakes, provides a particularly favorable opportunity to study the temporal characteristics of microbial mat development. In Lake Vanda, a punctuated rise in lake level has been well documented since 1973 [11], which allows us to construct a precise time-series of inundation. In addition, the upper waters of this lake are well mixed by convective processes [12] and the water column is very clear [13]. This means that conditions for microbial development in the zone inundated by lake level rise are not confounded by depth-related changes in water chemistry or by steep light gradients. The mats are internally laminated [14], and there is good evidence from other nearby lakes to suggest that these laminae record annual growth [5], allowing cross-validation of estimates of mat age. In this contribution, we present a detailed examination of the microbial mats in Lake Vanda, focusing on those that can be dated by recent changes in lake level. We use measurements made at similar depths and locations in 1998 and 2010 to describe temporal changes in mats and discuss these results in the context of the timescales of environmental change and ecosystem response.

## 2. Methods

### 2.1. Study Site

**Figure S1:**
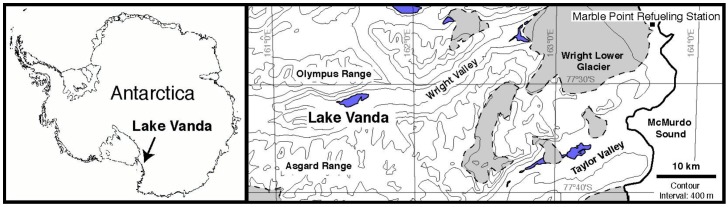
Map showing the location of Lake Vanda in the Wright Valley, McMurdo Dry Valleys region of Antarctica.

**Figure S2:**
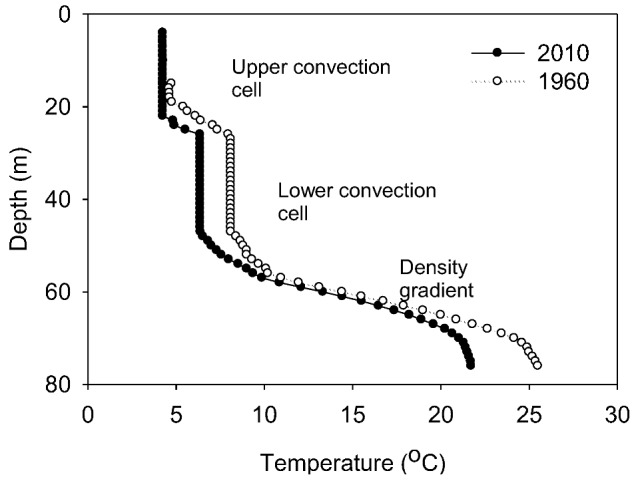
Water column structure in Lake Vanda in 1960–1961 and in 2010–2011. 1960–1961 data [15] were offset vertically by 11 m to allow for the increase in lake level. 2010 data were obtained with a YSI 6600 Sonde (http://www.ysi.com) in November 2010. The identification of upper and lower isohaline and isothermal convection cells overlying a continuous density gradient is consistent with other published profiles of the water column [12].

Lake Vanda lies in the Wright Valley, one of the MDVs in southern Victoria Land (77.52° S 161.67° E; Figure S1). It occupies a closed basin, and in 2010, it was a little over 75 m deep, with a perennial ice cover 3.5 to 4.0 m thick, though for several weeks each summer, the ice around parts of the lake shore melts to produce a discontinuous, open-water moat. The lake has an unusual physical structure, with temperature increasing with depth, from 4 °C just below the ice cover to >20 °C [15] (Figure S2). This inverse temperature gradient is due to solar heating and is stabilized by increasing solute content with depth [12]. Temperature and conductivity increase in two steps between 4 and 45 m, these being separated by a pycnocline at 20–25 m. These two upper steps are individually mixed by thermohaline convection [12], while below 45 m, there is a continuous gradual increase in temperature and conductivity.

The ice cover transmits 15%–20% of incident photosynthetically active radiation (PAR), heavily biased to wavelengths below 550 nm [14]. The water of the lake is also extraordinarily clear, with a vertical extinction coefficient for a downwelling PAR of 0.06 m^−1^ [13].

Several investigations of the plankton of Lake Vanda [16,17,18] report very sparse phytoplankton with a deep maximum of chlorophyll-a and photosynthesis at 55–65 m within the steep basal pycnocline [19]. Vincent and Vincent [16] provided strong evidence that phytoplankton abundance is limited by phosphorus supply. Zooplankton in the lake are restricted to five categories of ciliates and sparse numbers of the rotifer *Philodina gregaria* [20].

In contrast, benthic microbial mats are abundant and attain high biomass in Lake Vanda [4,14]. In 1980, these mats were reported to comprise mostly cyanobacteria, particularly species of *Phormidium* and *Lyngbya*, with pennate diatoms (species of *Navicula, Nitzschia, Caloneis* and *Stauroneis*) and occasional strands of moss [21]. In 1980, mats at a depth of 8.7 m (allowing for depth increase; see below; this would correspond to a depth ~18 m in 2010) were described as 2–5 mm thick and comprising a thin, orange-brown laminated mat overlying rock [21]. From 12–31 m (~21–40 m in 2010), Love *et al.* [21] described conical pinnacles with peaks 20–50 mm high and bases 30–50 cm in diameter, containing sub-mm thick laminations and calcite granules. Photographs included by Love *et al.* [21] suggest that these quoted sizes relate to the larger pinnacles and that smaller cones/pinnacles just a few mm in size were also present. 

Photosynthetic activity has been demonstrated in benthic mats to at least 40 m [14], and modeling studies suggest that these benthic communities are responsible for the bulk of primary production in Lake Vanda [5]. Hawes and Schwarz [14,22] noted that the microbial mat communities growing in the seasonally melted “moat” regions around the edges of MDV lakes are quite distinct from those under perennial ice cover, lacking laminations or distinct vertical structure and adapted to high irradiance.

### 2.2. Lake Level Rise

**Figure 1:**
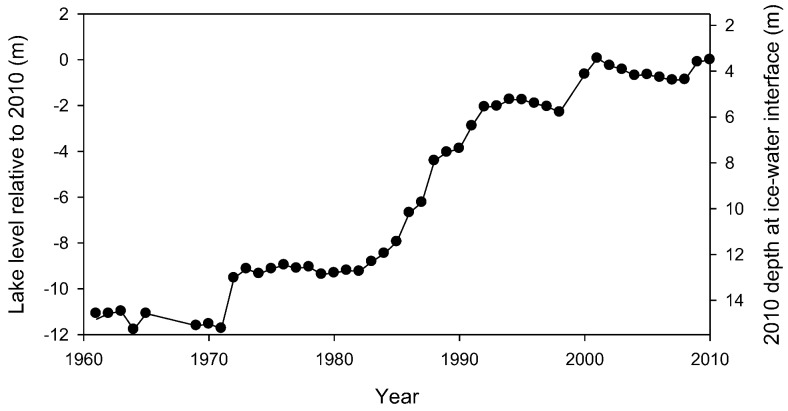
Change in Lake Vanda water level over time. Values on left scale are based on actual level measurements relative to fixed lakeside benchmarks from 1969 onwards and prior to this on reports of lake depths and water column structure. They are normalized to level in 2010 and show, for example, that in 1990, the lake was approximately 4 m shallower than in 2010. The right scale refers to the 2010 depth at which the underside of a 3.75 m thick ice cover was located in each year and shows, for example, that substrate at 10 m depth in 2010 had been below the lake ice cover since approximately 1987.

Having no outflow, the depth of Lake Vanda is dependent on the balance between inflow via the Onyx River and ablative/evaporative losses from the lake surface [11]. A robust record of lake level for Lake Vanda has been maintained since 1969 ([11], http://www.mcmlter.org), and this, combined with comparison of depth *vs.* temperature profiles that date back to 1960 [15], allows a 50-year record of lake level change to be constructed (Figure 1). Over the past 40 years, the water balance has favored accrual, and Lake Vanda has risen in level, though there have also been several multi-year periods of stable lake levels. Comparison of temperature profiles from 1960 and recent years shows that the thickness of the lower convection cell and the deep density gradient have not changed and that water column discontinuities are at the same position relative to the bed of the lake. This suggests that level increase has been largely confined to an expansion of the upper convecting cell (Figure S2). 

The increase in lake level constrains the time that specific depths have been below the 3.5–4 m thick perennial lake ice and, thus, the time available for growth of benthic microbial communities. For example, Figure 1 shows that the lake bed at 6 m in 2010 had been below the ice for ~18 years, at ~10 m for approximately 24 years and at ~14 m for 38 years. 

### 2.3. Sampling

Water column temperature profile in November 2010 was obtained with a YSI 6600 Sonde. Sampling of microbial mats was by SCUBA divers operating through access holes made in the lake ice. The access holes were made at similar locations in the lake (77° 31.63´S, 161° 36.33´ E) in both 1998 and 2010. Principal microbial mat sampling depths were those that, in November–December 1998, were at 4, 6, 8, 10, 12, 16, 20 and 24 m. In November–December 2010, we resampled at 6, 10, 14, 18, 22 and 26 m depth, which, allowing for the 2 m lake level rise that had occurred between samplings, were approximately the same locations as the 1998 sampling. In addition, in 1998, samples were taken from the moat region of the lake at depths of 1 and 2 m and under ice at 6 and 10 m.

### 2.4. Macroscale Morphology

**Figure 2:**
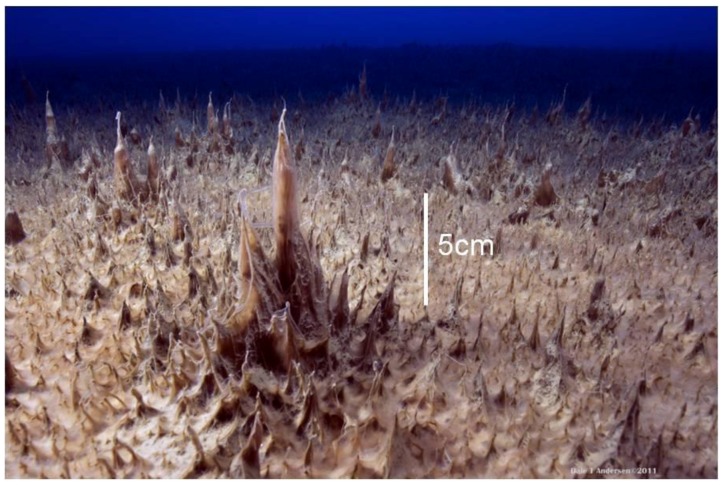
Oblique image of a microbial mat community with well developed pinnacles rising from a prostrate background in Lake Vanda at 18 m depth in December 2010. The scale bar refers to the large central pinnacle.

Benthic communities were comprised of undulating sheets of prostrate microbial mats from which pinnacles grew (Figure 2). The thickness of the prostrate mat to the underlying coarse sediments was measured using a sharpened, graduated rod. To obtain pinnacle height, a 0.4 m diameter circular quadrat was dropped haphazardly onto the mat surface, and pinnacles were measured as height above the prostrate mat surface systematically along a series of transects within the quadrat to ±1 mm by SCUBA divers using a graduated rod. The number of pinnacles measured in each quadrat varied with depth, with a minimum of 40 and a maximum of 120. Three quadrats were cast at each depth, and overall median and quartiles (25 and 75 percentiles) of pinnacle height were calculated after pooling all data from each depth.

### 2.5. Species Composition

Microbial mats in the upper part of Lake Vanda have an upper layer that is cohesive and contained all pigmented and some unpigmented laminae, as well as the vast majority of photosynthesis and aerobic respiration [14]. In 1998, samples of this cohesive layer were taken from the principal sample depths. Triplicate samples were cut from the mat at each sampling depth using a sharpened 60 mL syringe and placed in a darkened box for return to the ice surface. They were frozen and returned to New Zealand for microscope analysis. Examination was at magnifications of up to 1,000× and % abundance of recognizable morphotypes scored by counting subsamples from homogenized active layers, with a minimum count of 400 individuals. Cyanobacterial filaments and individual diatoms were considered a counting unit, this tending to overstate the absolute abundance of the latter. No coccoid cyanobacteria were encountered in these counts, and though we have identified these in other Antarctic microbial mats where they form colonies [23], we cannot exclude isolated coccoids in Lake Vanda mats.

In 2010, the differently pigmented zones within the mats were examined on site immediately after collection, again at magnifications of up to 1,000×. In addition, at depths of 10 and 18 m, mats were carefully dissected and the relative abundance of morphotypes within different zones evaluated. One sample was examined from each zone, and morphotypes were scored for abundance on a scale of 1 (present in some fields), 2 (present in all fields) and 3 (many trichomes in all fields). Morphotype identifications were made as described by Sutherland and Hawes [24], but cyanobacterial morphotypes from 1998 are reported only to a level where we can be confident that similar identities would have been ascribed in 2010. Evaluation of taxonomic status of cyanobacteria collected in 2010 by molecular approaches is ongoing and will be presented separately. For the current study, we consider only the consistency of morphotypes by depth, by location within the mat and over time.

### 2.6. Biomass and Pigments

In 1998, a microbial mat was cut out by divers using a knife, and the active layer was transferred to darkened boxes and returned to the surface (n = 5). Known-area samples were cut from these samples using a 25 mm diameter coring device. For depths of 4 to 12 m in 1998, the full mat thickness was taken as a single cohesive unit, whereas at greater depths, non-cohesive organic material was found below the upper cohesive and pigmented layer, but was not collected. Samples were collected haphazardly, though areas with unusually large or complex surface topography were avoided to optimize replication. 

In 2010, divers cut through the mats to the underlying coarse sediment with a 38 mm diameter, sharpened plastic ring. The resulting core, which included the upper cohesive layer and any underlying organic material, was transferred to an individual container and returned to the surface. Five haphazardly selected replicates were taken at each depth, and once again, unusually large microbial features were avoided. 

Prior to analysis, samples were stored for up to 2 months at −20 °C, then freeze-dried. Weighed aliquots were taken for analysis of loss of mass on acidification (LoAc—inferred as calcite content) and loss of mass on ignition (LoI), carbon and nitrogen content (also phosphorus in 1998) and chlorophyll-a. In 2010, aliquots were additionally taken for determination of other acetone-soluble pigments. Methods are fully described in Hawes *et al.*, [10], but briefly, LoAc was determined by weight change before and after addition of 10% HCl to oven dried samples (60 °C), ash was determined after subsequent combustion at 450 °C for three hours and LoI calculated as oven-dry mass less LoAc and ash. In both years, LoAc and ash are expressed as a percentage of dry mass. Organic C and N contents were measured on weighed aliquots (after acidification) using a Carlo Erba Automated CHN analyzer. Organic P was estimated as reactive P after oxidation by acid persulfate. Because the sampling method used in 2010 included accumulated organic material to the underlying gravels, elemental contents are reported as mg cm^−2^, while because only the cohesive pigmented upper layer was sampled in 1998, they are expressed as % dry weight.

For chlorophyll-a and other acetone-soluble pigment analysis, aliquots were extracted by ultrasonication (15–20 W for 30 seconds) in ice-cold 95% acetone and left in the dark for 24 h to complete extraction. After clarification by centrifugation, chlorophyll-a concentration was determined by spectrophotometry, without acidification [25]. In 2010, three extracts were randomly selected from the five replicates for each depth, transferred to a sealed glass vial and stored at −80 °C under nitrogen for up to 50 days before a more detailed analysis of pigments was undertaken by High Performance Liquid Chromatography (HPLC) [10]. A Dionex HPLC system, with PDA-100 diode array detector (300–800 nm), separated pigments according to the chromatographic method of Zapata *et al.* [26]. Pigments were quantified by absorption at 436 nm, with calibration by reference to commercially available standards.

Statistical treatment comprised calculation of means and standard deviations, and comparisons used targeted 1-way ANOVA, where appropriate. Sigmastat 3.5 (www.systat.com) was used for all statistical analyses.

### 2.7. Variable Chlorophyll-a Fluorescence

We examined vertical sections of mat samples returned to the surface using a pulse amplitude modulated (PAM) fluorescence analysis [27] to provide an estimate of the distribution of potential photosystem II (PSII) electron transport activity. Vertical sections, each approximately 5 mm thick, were cut with a scalpel from mat samples from 6, 10 and 18 m depth, laid on their sides and examined using a Walz Imaging-PAM fluorometer (Walz Mess- und Regeltechnik, Germany). Imaging PAM fluorometry allows resolution of the distribution of pigment systems in microbial mats and the activity of photosystem II (PSII) in two dimensions [10,27,28]). The instrument was fitted with a lens imaging an area of 30 × 23 mm and the saturation pulse method used to determine minimum (*F*_o_) and maximum (*F*_m_) fluorescence yields of dark-adapted samples to a pulsed blue light. From these values, we calculate variable fluorescence, *F*_v_, as (*F*_m_ – *F*_o_) and derived the maximum quantum yield of PSII as *F*_v_/*F*_m_. For a detailed description of the saturation pulse method, see Schreiber [27]. Care was taken to avoid exposure of samples to bright light after collection, and all measures were taken after at least 30 minutes of darkness. The measuring light of our instrument was provided by blue LEDs and peaked at 450 nm. While cyanobacteria often absorb this wavelength only weakly, we considered the wavelength to be acceptable given the spectral qualities of light under Lake Vanda ice, which contains almost no red and is dominated by blue and green wavelengths [14]. However, the method may be biased towards diatoms over cyanobacteria, since the former absorb blue light well, and results must be interpreted with care. Prior to use, the imaging system was adjusted using a fluorescent plastic target to ensure that equal fluorescent yield was returned from all parts of the image in the focal plane of the lens, according to manufacturer’s recommendations.

The Imaging-PAM outputs fluorescence parameters as false-color images, the color scale ranging from red through violet indicating ranges of 0 to 1. Images of dark adapted fluorescence yield (*F*_o_) were taken as a proxy of the distribution of chlorophyll-a. In this case, the images are returned in arbitrary units that can be quantitatively interpreted only within individual images. Images of the maximum photochemical yield (*F*_v_/*F*_m_) are dimensionless and can be compared between images. The Imaging-PAM instrument also records images of samples under red and near infra-red illumination and approximates chlorophyll-a absorption as A = 1 − (R/NIR). To ensure that absorption images were meaningful, care was taken to adjust the intensity of the R and NIR emitters such that a white calibration target returned an absorption of zero.

### 2.8. Microelectrodes

The distribution of net photosynthetic activity within microbial mats at 18 m was also inferred from the profiles of dissolved oxygen in mats and the immediate overlying water column. These profiles were measured by divers, *in situ*, using a Clark-type underwater O_2_ microelectrode [29] (Unisense—www.unisense.com) attached to a manually operated micromanipulator mounted on an aluminum post [28]. Constraints on divers at 18 m depth under lake ice and instrument failures restricted the number of profiles that could be obtained. An underwater picoammeter (Unisense PA 3000U) provided the polarization voltage for the O_2_ microelectrode, which had an outside tip diameter of 50 µm, a 90% response time of ~1 s and a stirring sensitivity of ~2%. The diver used the micromanipulator to move the electrode in 0.2-mm increments normal to the mat surface from a position above the diffusive boundary layer (DBL) to a depth of 6–7 mm into the mat. The position of the mat surface was estimated by the diver and confirmed by a break in the dissolved O_2_ profile. The diffusive flux (J) of O_2_ from the microbial mat into the overlying bottom water was calculated from the measured steady-state O_2_ gradients (dC/dz) within the diffusive boundary layer (DBL), according to methods fully described in Vopel and Hawes [28]. 

## 3. Results

Throughout this section, all depths are referenced to water level in 2010. Our water column profile (Figure S2) identified upper and lower isohaline and isothermal convection cells overlying a continuous density gradient, and the locations of the discontinuities are consistent with other published profiles of the water column [12], confirming that lake level rise had been achieved by an increase in the thickness of the upper convection cell.

### 3.1. Macroscale Morphology

**Figure 3:**
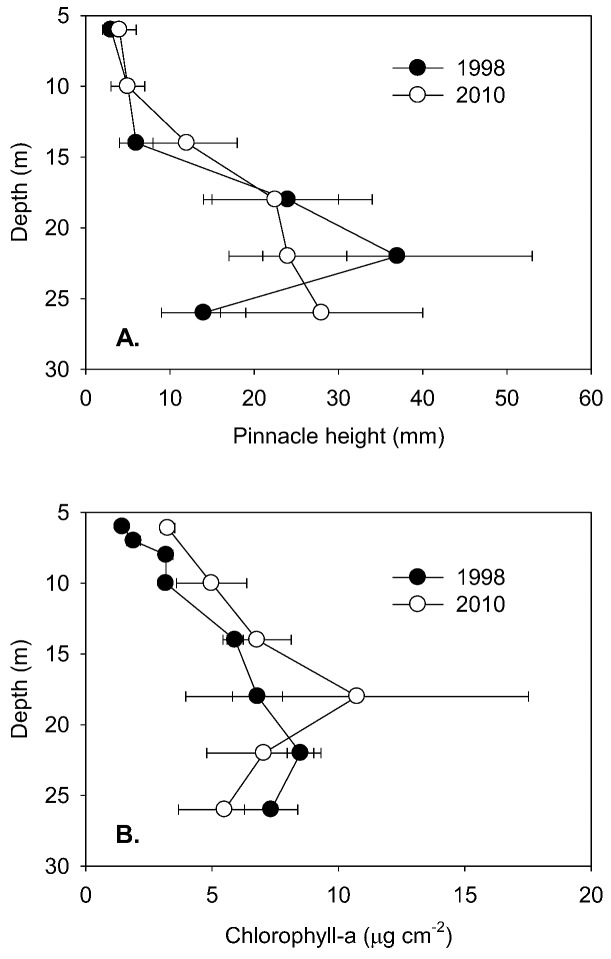
A. Pinnacle heights (median and quartiles, n > 100) at selected depths in Lake Vanda in 1998 and 2010. B. Concentrations of benthic chlorophyll-a (mean and s.d., n = 5) at various levels in Lake Vanda in 1998 and 2010. Both profiles are referred to depth in 2010.

**Figure 4:**
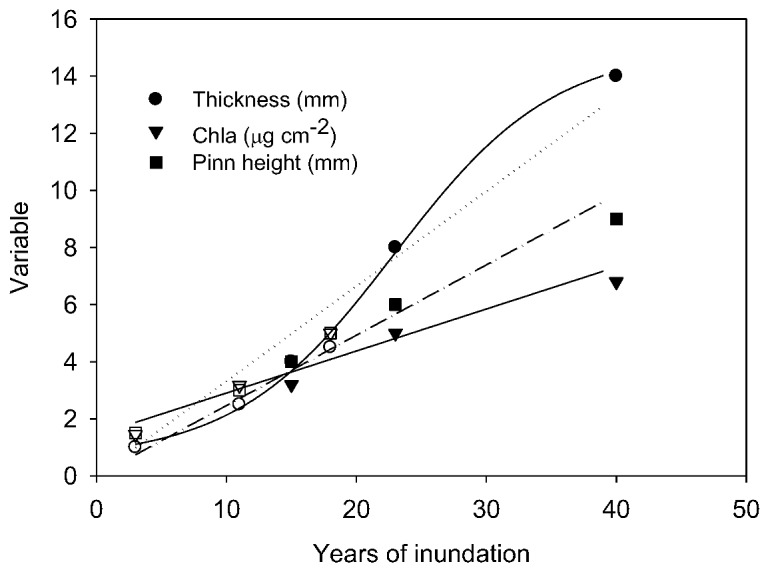
Relationships between median pinnacle height, areal chlorophyll-a content and thickness of flat mat from a range of depths, expressed as time under ice. Solid symbols represent samples taken in 2010; open symbols in 1998. Linear regression models are fitted to each variable for all samples and are significant at *p* < 0.01. For the mat thickness, a sigmoid curve provided a better fit (r^2^ = 0.998 *cf.* r^2^ = 0.959 for the linear model). Pinn height—pinnacle height.

To the maximum depth sampled, the lake floor was covered by a flat to undulating mat from which pinnacles of varying height emerged (Figure 2). Mat thickness increased with depth on both sampling occasions and, over the 6–18 m depth interval, also increased between samplings (Table 1). The rate of increase in thickness increased with depth to 18 m, with 12 years of growth producing an increased thickness of 0.3–1.1 mm y^−1^ (Table 1). Pinnacle height also tended to increase with depth into the lake (Figure 3A), with a discontinuity in the rate of increase at 10–15 m and an apparent maximum height reached at ~22–26 m. Pinnacle height, even after log-transformation, was highly variable and not normally distributed, and a Kruskal-Wallis ANOVA by ranks yielded no significant differences (at *p* = 0.05) at specific depths between 1998 and 2010 samplings. In 2010, ANOVA by ranks clustered pinnacle height into three depth-groups, from 6 and 10 m (group median height 5 mm) and from 18, 22 and 26 m depth (group median height 24 mm), while those from 14 m (median height 12 mm) fell significantly between these groups. By plotting thickness and median pinnacle height against time under ice, where this last could be estimated, a consistent trend of a net increase over the early period of inundation was apparent (Figure 4). A highly significant linear increase in pinnacle height of ~0.3 mm y^−1^ (r^2^ = 0.97, *p* < 0.01) was seen in the known-age part of the water column, that is, from 6–14 m. For thickness, a significant linear relationship was also apparent (r^2^ = 0.80, n = 6, *p* < 0.05), suggesting a rate of growth of 0.14 mm y^−1^. A curve fitting exercise comparing a range of models for these time-dependent data suggested that there was a tendency for rates of increase to decline after several decades (Table S1), though the estimates of initial rates of increase were very similar to those obtained from linear fits.

**Table 1:** Characteristics of microbial mats along a depth profile in Lake Vanda in 1998 and in 2010. Depths are referred to 2010 lake levels.

Depth in 2010 (m)	Approximate age in 1998 (y)	Total thickness of flat mat 1998 (number of laminae)	Total thickness of flat mat 2010 (number of laminae)	Annualized rate of increase in thickness (number of laminae)
6	6	1 mm (2–3)	4 mm (12)	0.3 (0.85)
10	10	1–2 mm (7)	8 mm (18)	0.5 (0.92)
14	28	4 mm (>12)	14 mm (>20)	0.8 (-)
18	>50	5 mm (>14)	18 mm (>20)	1.1 (-)
22	>50	>15 mm	25 mm	- (-)
26	>50	>50 mm	>45 mm	- (-)

### 3.2. Internal Morphology

In both 1998 and 2010, mats contained up to four zones distinguishable by pigmentation. An orange-brown zone overlay green and then purple zones, which, in turn overlay a non-pigmented zone. The shallowest mats did not contain green and purple zones in 1998, but at 6 m depth in 2010 and 10 m depth in 1998, all zones were present. On a finer scale, each zone contained multiple laminae, which varied in thickness from several hundred microns to more than a millimeter, with some undulations in thickness within individual laminae. Laminae were defined by bands of fine sediments separating otherwise organic-rich, mucilaginous material. Sediment bands varied from barely discernible to up to 1 mm thick, and a particularly dense band of sediment was seen across depths at the base of the 9^th^ lamina from the surface.

In 1998, at the shallowest depth sampled (equivalent to 6 m in 2010), the mat was 1 mm thick and contained only an orange-brown zone, comprising 2-3 recognizable laminae. At the same location in 2010, the mat had increased in thickness to 4 mm and contained all color zones. Four orange-brown laminae, each approximately 0.2 mm thick, capped 6-7 green-purple laminae of ~0.5 mm thickness, giving a total of ~12 organo-sedimentary laminae of which ten were pigmented. 

At a (2010) lake depth of 10 m, a 2 mm thick mat was present in 1998, with 7 recognizable laminae (3–4 orange, 3–4 green-purple). In 2010 the number of laminae at this location had increased to ~18, with four orange laminae overlying 6–8 green and purple laminae, fading to ~4 colorless laminae and becoming increasingly difficult to resolve. These observations showed that the subsurface laminae and zones were continuing to thicken after new laminae had formed.

At water depths exceeding 14 m, the clearly laminated and pigmented zones overlay an increasingly thick and less distinctly laminated mix of organic material and fine silts. Once the lamina count exceeded 20, counting became difficult. However, wherever counting was feasible, approximately 10–12 more laminae were present at any given depth horizon in 2010 than in 1998, consistent with the accumulation of one lamina per year (Table 1). The picture that emerges is of mats accumulating by annual lamina accrual, with the characteristics of laminae evolving from thin, orange-brown to thicker, purple or green and, ultimately, to colorless as they are progressively overgrown.

### 3.3. Species Composition

Cyanobacteria formed the matrix of mats, and the dominant morphotypes on both samplings, across all lake depths and locations within the mats, were identified as belonging to the genus *Leptolyngbya*. In 1998, *Leptolyngbya* comprised 40%–50% of the total counts at all depths, with the remainder ascribable to *Phormidium* (5%–20%) and *Oscillatoria* (2%–5%). In 1998, the 6 m mat also contained 5% *Nostoc*, a genus not seen in 2010, but which was also common (36 and 10% relative abundance, declining with depth) in the samples from within the moat of Lake Vanda. 

In 2010, four morphotypes of *Leptolyngbya* were distinguished on the basis of cell dimensions. Morphotype diversity appeared to decline with depth in the lake, though our data do not allow robust statistical comparisons. At 10 m, three *Leptolyngbya* morphotypes were common across all color zones, and one other was present, whereas at 18 m depth, only one morphotype (1–2 µm wide, square cells with no constriction) was dominant in all color zones (Table S2).

Orange-brown surface zones consisted almost exclusively of *Leptolynbya,* usually oriented vertically and taking the form of ropes and columns of intertwined trichomes within laminae and tending to extend vertically out of the surface of the mat as tufts. Trichomes showed a tendency to orient horizontally at lamina interfaces. The green zone, if present, was immediately below the orange layer and comprised two or three laminations. *Leptolyngbya* was, again, overwhelmingly dominant. *Oscillatoria* cf. *sancta*, *Phormidium* cf. *autumnale*, *P.* cf. *murrayi* and a *Pseudanabaena* sp also occurred in the mats, but always at low abundance. Here, as in the orange-brown zone, *Leptolyngbya* trichomes tended to be oriented vertically and were intertwined. The transition from green to purple coincided with an increase in *Phormidium* and *Leptolyngbya* morphotypes that were visibly full of phycoerythrin. In the purple zone, trichomes were more widely spaced and less well-oriented than higher in the mat (Table S2). 

Diatoms, notably Navicula muticopsis forma murrayi (14%–28% of total diatoms), Diadesmis contenta and D. contenta var. parallela (2%–9%), Hantzschia amphioxys var. maior (7%–34%) and Muelleria peraustralis (7%–28%) were common across depths, though less so close to the ice cover, and a coccoid eukaryote, tentatively identified as a species of Chrysosococcus, was at times frequent. No clear differences were evident in diatom relative abundances between depths, and methodological differences preclude a quantitative comparison between years. Similar species lists and degrees of dominance were, however, seen across years.

### 3.4. Biomass and Pigments

Quantitative comparisons between 1998 and 2010 are most robust for chlorophyll-a, where analytical methods were similar in the two years. In both years, chlorophyll-a increased with lake depth to a maximum at 18 m (Figure 3B), and in all but the deepest sample, chlorophyll-a concentrations were higher in 2010 than in 1998. However, only at 6 and 10 m were these increases statistically significant (ANOVA, *p* < 0.05). Within the 6–14 m depth band, chlorophyll-a increased steadily with age (Figure 4) equivalent to an annualized rate of 0.27 µg cm^−2^ y^−1^ (linear regression, r^2^ = 0.90, n = 6, *p* < 0.01). The intercept of chlorophyll-a with age below ice is not zero, and this most likely reflects that the under-ice mats are developing not from barren ground, but from mats that were previously in the seasonally frozen marginal zone [14]. Comparison of other curve fits to this small data set suggested that the rate of increase in chlorophyll-a tended to decrease after some decades (Table S1), though similar rates of increase during the first 30 years after inundation are indicated. HPLC analysis of pigments in 2010 showed similar pigment profiles, with some pigments showing proportional changes between lake depths (Table 2). Two cyanobacterial pigments, myxoxanthophyll and nostoxanthin, showed a decline in relative abundance with depth to 14 m, whereas three pigments associated with diatoms, chlorophyll-c, diadinoxanthin and fucoxanthin, increased to 14 m. Two taxonomically widespread carotenoids, β-carotene and canthaxanthin, all tended to decline gradually with depth and reached minima at 26 m. Of the recognized chlorophyll-a derivatives, when expressed as ratio to chlorophyll-a, an allomer, phaeophytin and phaeophorbide increased with depth to 22 m, whereas chlorophyllides showed only a slight increase in relative abundance with depth. No bacteriochlorophylls were observed, though the technique used detects these when they are present.

**Figure 5:**
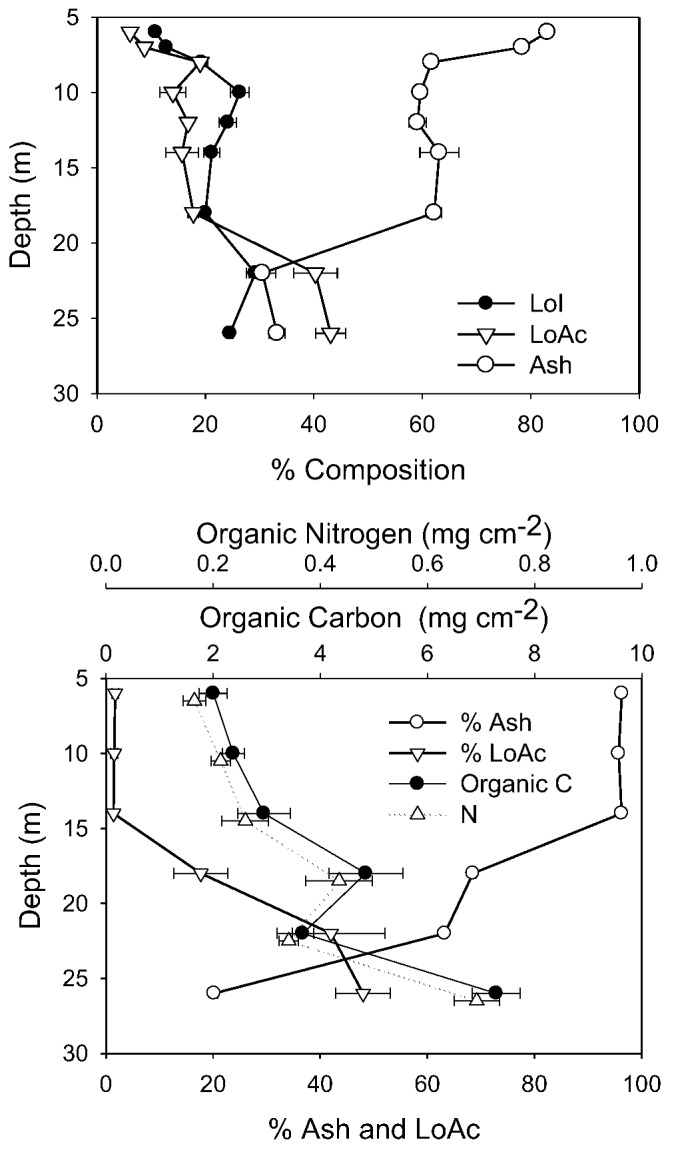
Biomass-related variables from 1998 and 2010. In 1998 (top) only % composition was measured, and all depths refer to the “active layer” of mats only. In 2010 (bottom), all depths, except 26 m, comprised cores to the underlying coarse sediments and were normalized to unit area. The 26 m sample represents only the active layer. In each case, the mean and standard deviation are plotted (n = 5).

It is not possible to compare directly other biomass determinants between 1998 and 2010 due to slightly different sampling and analytical methods. However, in both years, the compositional changes with depth showed similar trends, and the differences between years were strongly associated with the ash content. In 1998, ash content was very high (90%) immediately below the ice, declining to ~65% from 8 to 18 m, then falling again to 30% at 22 m and below (Figure 5). In 2010, ash content was also very high immediately under the ice (99%), but on this sampling remained high to 14 m (Figure 5). However, as in 1998 ash content declined at greater depths, falling to 60% at 18 m and 20% at 26 m. The decline in % ash at 22–26 m depth was balanced by an increase in %LoAc, which in both years, reached 40%–50% here. Microscopy in 2010 supported these composition data, as shallow depth mats contained substantial amounts of both fines and sands, whereas at greater depths, sand was rare, and mineral inclusions were primarily small amounts of fine clay and precipitated calcite.

In 2010, when area-specific concentrations were measured, carbon and nitrogen both increased near exponentially to 18 m depth, closely paralleling the increase in chlorophyll-a (Figure 3), though at 22 m depth, both C and N were anomalously low. Atomic ratios of C:N in both 1998 and 2010 were consistent across depths at 12–16 (1998) and 12–14 (2010). In 1998, N:P atomic ratio averaged 40:1 (range 33–54:1) across the 6–26 m depth range, with no clear depth-related pattern.

**Table 2:** Relative abundance of selected pigments determined by high-performance liquid chromatography (HPLC) at the principal sampling depths in November 2010.

	6 m	10 m	14 m	18 m	22 m	26 m
Myxoxanthophyll	5.3	2.6	1.3	1.4	1.1	1.9
Nostoxanthin	1.8	1.0	0.5	0.6	0.7	0.9
Chlorophyll-c	0.4	2.1	3.7	3.9	4.1	3.9
Diadinoxanthin	0.2	1.2	2.0	2.2	2.4	1.0
Fucoxanthin	2.7	6.4	10.9	12.0	12.5	10.0
β-carotene	1.2	1.0	0.5	0.8	0.7	0.7
Canthaxanthin	1.6	1.2	0.9	0.9	0.9	0.6
Chl-a allomer	4.8	8.9	23.5	21.3	26.1	19.7
Phaeophorbide-a	3.0	11.0	25.4	29.3	30.6	23.3
Chlorophyllide-a	13.1	18.1	19.8	17.0	28.5	18.3
Phaeophytin-a	1.1	6.3	18.5	15.1	14.7	4.5

For the upper seven pigments, results are expressed as % total pigment, whereas the four chlorophyll-a derivatives at the bottom of the table as ratio to chlorophyll-a (×100).

### 3.5. Variable Chlorophyll-a Fluorescence

**Figure 6:**
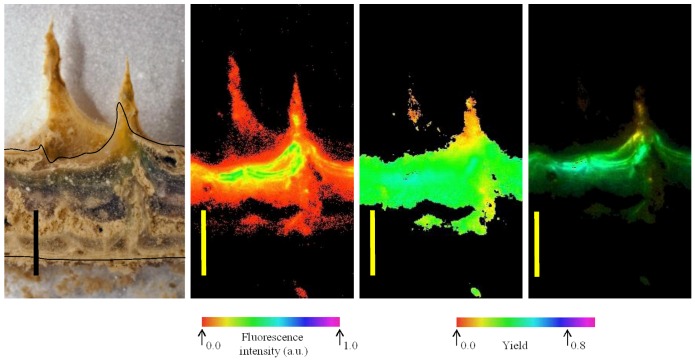
Distribution of fluorescence intensity (minimum fluorescence yield [*F*_o_]—second image from left) and maximum yield of photosystem II (the maximum quantum yield of PSII [*F*_v_/*F*_m_]—third image) in part of a vertical section of a mat from 10 m depth. At far right is a maximum fluorescence yield (*F*_m_)-weighted yield image, where the false color represents the yield and the intensity represents *F*_m_. Color scales below fluorescence images represent imaging pulse amplitude modulated (PAM)-derived false-color values. At far left is a photographic image of the mat section at the same scale on which the annotated line shows the outline of the cut surface. Scale bars are 5 mm.

**Figure S3:**
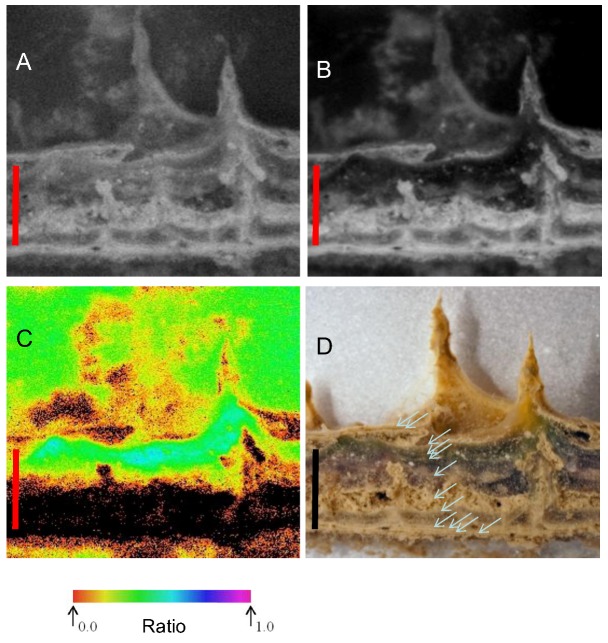
“Absorption” of red light, a proxy for the distribution of chlorophyll-a, by the microbial mat in Figure 9, collected at 10 m depth. Image A was taken in near infra-red light, image B in red light and image C is a false color image of chlorophyll-a absorption calculated as (1-R/NIR). Image D is a photograph of the mat cross section, and arrows indicate the locations of sediment-rich laminae. Scale bars are 5 mm, and color scale indicates 1-R/NIR.

Imaging of variable chlorophyll-a fluorescence provided insights into both the location of areas where pigments were most abundant and of potential electron transport capacity. Four images of a vertical section of microbial mat from 10 m are shown in Figure 6. At the left, a photograph of the mat shows the orange, green and pink-dominated zones overlying a near-colorless underlayer; the adjacent image shows how *F*_m_ is distributed primarily into the laminae below the surface. Note that the cut surface of the section is indicated in the left hand image, mat components behind the cut surface are further from the camera and fluorescence yield cannot be directly compared. The third image from the left indicates that the yield of PSII under experimental conditions was rather evenly distributed, with *F*_v_/*F*_m_ of 0.4–0.5, with the possible exception of the orange zone, where *F*_v_/*F*_m_ values of ~0.25 were observed. The right hand image combines *F*_m_ and *F*_v_/*F*_m_, in that the intensity of the image is scaled to *F*_m_, while the color is scaled to *F*_v_/*F*_m_. These images together show that potentially active photosystems are found in all of the pigmented layers and, indeed, that the highest values of *F*_v_/*F*_m_ occurred within the purple and green zones, rather than close to the mat surfaces. Images also show bands of high pigment concentration at intervals below the mat surface, and comparison with the photograph shows how these are associated with lamina boundaries. Absorption images support the view that chlorophyll-a is distributed well down into the deeper laminae of the mats, indicated by absorption of red light, but not near infra-red light (Figure S3).

**Figure S4:**
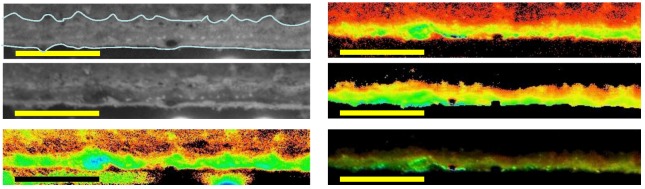
Vertical cross section of a microbial mat from 6 m depth. A and B are images obtained under infra-red and red light, and image C is the absorption, expressed as false color (as in Figure S3). Image D: distribution of maximal fluorescence intensity (*F*_m_). Image E: maximum yield of photosystem II (*F*_v_/*F*_m_). Images D and E are combined in image F, which indicates *F*_v_/*F*_m_ according to false color value and *F*_m_ as intensity. Color scales represent imaging PAM-derived false-color values. False color scales are as in Figure 9, and the horizontal bar indicates 5 mm.

**Figure S5:**
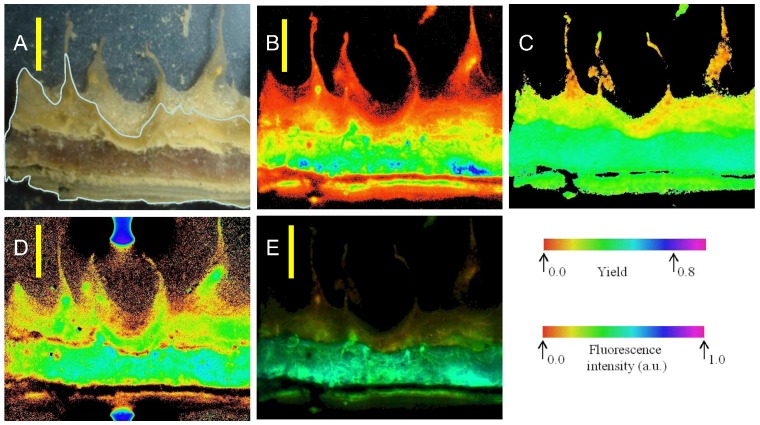
Vertical cross section of a microbial mat from 18 m depth. Image A is a photograph of the cross section, and the cut surface is outlined to indicate the part of each image that is in the focal plane; in all cases, the scale bar is 5 mm. Image B: distribution of maximal fluorescence intensity (*F*_m_). Image C: maximum yield of photosystem II (*F*_v_/*F*_m_). Images B and C are combined in image E, which indicates *F*_v_/*F*_m_ according to false color value and *F*_m_ as intensity. Color scales represent imaging PAM-derived false-color values. Image D is the “absorption” of the cross section, as defined in Figure S3, and indicates the distribution of chlorophyll-a.

Images of example mat sections from 6 m (Figure S4) show similar organization to Figure 6, though with a markedly thinner cross section. The 6 m images are slightly oblique, but when the cut surface is examined once again the distribution of fluorescence and absorptions indicate a concentration of pigments and PSII yield in the sub-surface laminae. Finally, images from 14 m depth samples show further evolution of this arrangement in a thicker mat section (Figure S5). Images at the left show the arrangement of pigmented zones and the localization of maximal chlorophyll-a absorption in the deeper laminae, while *F*_m_ and *F*_v_/*F*_m_ images indicate the maximum potential yield occurs in these deeper parts of the mats and that fluorescence is maximal at the lamina boundaries.

### 3.6. Oxygen Microprofiles

**Figure 7:**
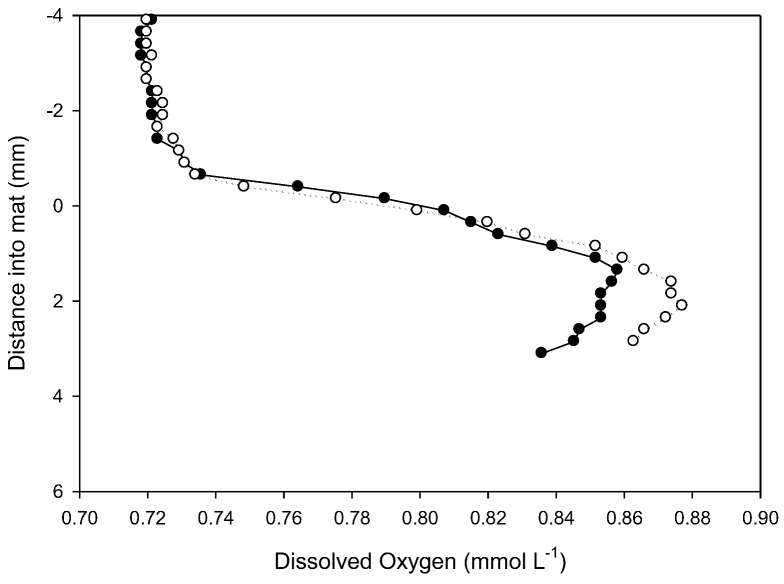
Two *in situ* profiles of dissolved oxygen within a prostrate mat at 18 m in Lake Vanda, obtained with microelectrodes. The vertical axis, in mm, locates the mat surface at 0 mm, with the water column as negative and the mat as positive displacements.

*In situ* dissolved oxygen microprofiles in prostrate mat at 18 m (Figure 7) were made at ambient temperature (4.2 °C, 602 µS cm^−1^ conductivity, pH 8.52 and a photon flux of 80 µmol m^−2^ s^−1^). As is common in Antarctic lakes, dissolved oxygen concentration in the water column exceeded atmospheric saturation [28]). Profiles show a rapid rise in dissolved O_2_ concentrations through the diffusive boundary layer and into the top part of the mat and a convex profile indicative of O_2_ production via photosynthesis to at least 4 mm. This is consistent with fluorescence analyses that show photosynthetic potential extending into the older laminae. Penetration into the mat was insufficient to document the activity of the full thickness of the microbial mat. Estimation of areal oxygen evolution rate from the concentration gradient in the DBL of Figure 7 yields a rate of 0.3 to 0.4 µg oxygen cm^−2^ h^−1^. This is of a similar magnitude to that reported earlier for this lake of −1 to +3 µg oxygen cm^−2^ h^−1^ from darkness to light saturation using *in vitro* incubation methods [14]. 

## 4. Discussion

Existing models of the growth and lamination of prostrate microbial mats in the MDV lakes are based on the accumulation of a new organic-rich layer during summer photosynthetic growth and the laying down of a sediment-rich layer during winter [5,6]. The increase in the number of laminae at specific locations in Lake Vanda between 1998 and 2010 was sufficiently close to 12 to support the view of these laminae as annual alternations between sediment deposition and mat growth. Our data suggest that mats tend to contain 2–3 laminae less than the number of years they have been within the main body of the lake, suggesting that annual banding does not occur within the seasonally frozen moat and becomes evident only 2–3 years after mats have emerged under the ice.

Vertical lamination is a characteristic of accreting microbial mats in clastic sedimentary environments. It is typically viewed as a consequence of filamentous cyanobacteria and diatoms moving through and overgrowing episodic sedimentary events and binding sediments in extracellular materials [2,30]. Lamination on an annual timescale, as seen in MDV lakes, is consistent with the seasonal rather than diel photoperiod at high latitude and the short pulse of flowing water and, hence, sediment influx during January [5,24]. Variability of the amount of sediment within laminae most likely reflects variations in summer sediment load, and unusually thick deposits are likely related to high sediment influx. In 2001–2002, a MDV-wide high flow event was recorded in response to unusually warm summer temperature [31], and we interpret the thick sediment layer seen across depths ~9 laminae down to be related to delivery of a large dose of sediment through this climate-driven event. It provides further mechanistic support for near annual nature of lamination, but caution is required in that sediment laminae can be expected to be less distinct during years with low sediment influx and are not completely reliable as annual phenomena. However, our observations show how changing climatic conditions can have complex effects on microbial communities, not just through rising lake levels and associated effects on irradiance, but also through changes to sediment flux. 

Gradual accumulation of microbial mat biomass through this process is evident by the increase in thickness, areal C, N and chlorophyll-a content with depth and years since emergence below the ice. Where it is possible to date mat initiation, predictable rates of increase in thickness, chlorophyll-a and pinnacle size were observed, though it is evident that mat dynamics during this early phase of growth, which may last for 3–4 decades, changes once mats reach maturity. At maturity, ongoing growth at the surface is likely to be increasingly offset by decomposition and compression of deeper laminae, though gradual accumulation of organic material and thickness appears to continue. Pigment per unit area can also be expected to saturate, as the effects of increasing biomass on self-shading will be compounded by the effects of reduced irradiance. Even if pigment biomass accumulated to light-limited status, it will still be expected to decline with depth within the mature mat community due to declining irradiance [24]. Indeed, we would expect that the ultimate, light-limited chlorophyll-a biomass that could be achieved will be declining over time in the deep, mature mats, as irradiance is reduced by increasing water depth. Thus, the response of deep communities to lake level rise can be expected to be opposite to those growing in new habitat, with slower growth and declining photosynthetically active biomass, though our data are insufficient to quantify any such change.

Microbial mats are clearly a sink for C, N and P in Lake Vanda at all depths. The rates of accumulation of C in sediments at depth contours of known age, in the shallow part of the lake where mats are still accruing towards saturation biomass, can be estimated from Figure 5 as approximately 390 mg m^−2^ y^−1^. This equates to an average of 104 µg oxygen cm^−2^ y^−1^ (using a photosynthetic quotient of 1:1) and extrapolating the very limited data on areal oxygen evolution presented here (0.3 µg oxygen cm^−2^ h^−1^ would generate 104 µg oxygen cm^−2^ in 14 days) suggests that such a rate of accrual is quite feasible given that sufficient irradiance is likely to be present for at least 100 days of photosynthesis. Using C:N and N:P mass ratios of approximately 14 and 90:1 measured here, this carbon accrual is equivalent to approximately 24 and 1.4 µg m^−2^ y^−1^ of N and P. Jones-Lee an Lee [32] provided data from which an annual external load of P to Lake Vanda can be estimated as 2.3 µg m^−2^ y^−1^, a value similar to that calculated for mat sequestration and suggesting that mats may be an important sink for nutrients entering the upper part of the water column from outside of the lake.

While annual accretion of thin laminae of microbial mat at the surface is supported by our data, a growth model solely based on accrual of a new layer is only partially supported. Increases in the thickness of subsurface laminae relative to the surface, shifts in pigment contents and species composition and ongoing photosynthetic capacity with higher maximum yield of PSII than surface laminae suggest that the buried laminae remain significantly active for many years and contribute to mat thickening and pigment content

In Lake Vanda, we found a shift in *F*_v_/*F*_m_, as well as pigmentation at the orange-pink transition. Shifts in pigment composition can result in alterations in apparent yields of photosystems to a given excitation wavelength [33], particularly in cyanobacteria, where phycobilins can be associated to PSII rather than PSI [34]. It is possible that the shifts in *F*_v_/*F*_m_ at the pink-brown interface are thus methodological and may also involve the relative ability of different pigment complements to respond to the blue measuring light and to connect to the PSII centers. 

Internal metabolic zonation is, however, a characteristic of microbial mats. Pearl and Pinckney [35] summarized how the consortium of microorganisms within a mat typically creates and exploits steep gradients of environmental conditions over mm length scales. Zonation of metabolism within typical temperate microbial mats sees oxygenic photosynthesis dominating surface zones on a diurnal basis, with anoxygenic photosynthesis and sulfide metabolism embedded in steep gradients of light, oxygen, pH, redox potential and sulfide concentrations [1,36], as also observed from mats in shallow Antarctic melt ponds [37,38]. However, such acute metabolic zonation does not appear to be common in the upper waters of MDV lakes during summer, where steep gradients of dissolved oxygen are not apparent [28]. The reasons for this are not clear, though Vopel and Hawes [28] did calculate low rates of oxygen consumption within mats and may also relate to perennially high ambient oxygen concentration in overlying waters that prevent hypoxia within surface sediments.

The shift towards dominance of pigment profiles by phycobilins over carotenoids with depth into mats and the corresponding change in appearance from orange (carotenoids) to green (phycocyanin) and pink (phycoerythrin) is consistent with acclimation to changing spectral characteristics inside the microbial mats. Irradiance becomes increasingly green and orange inside Lake Vanda mats [14], and such depth-in-mat related spectral adaptation is shown in other types of microbial mat [1,39], albeit reflecting a shift to sulfur bacteria rather than phycobilin-rich cyanobacteria. Internal zonation boundaries did not mark a shift in dominant cyanobacterial phototrophs, based on morphotype distribution or in photosynthetic process, since photosystem II was clearly active here. Indeed, profiles of dissolved oxygen in Lake Vanda mats show concentrations well above 100% saturation to extend through the orange layer and into the pink, and no bacteriochlorophylls were detected by HPLC pigment analysis. Chloroflexi 16S rRNA gene sequences have been detected in the Lake Vanda mats (Jungblut, personal observations). Ongoing research is using molecular techniques to determine whether taxonomic shifts undetectable by microscopy are occurring with these zones. 

Lake Vanda is, however, highly oligotrophic, and zonation within mats may reflect differential supply of internally recycled nutrients. Quesada *et al.* [7] reported elevated nutrient concentrations within microbial mats relative to bulk lake water in nearby Lake Hoare, and other polar microbial mats from the Arctic can be internally nutrient replete [40], assisted by potential microbial recycling and scavenging processes [41]. The paradigm of microbial mats acquiring, then efficiently retaining and recycling, nutrients has been advanced to explain this disparity. A potential “crossed resource gradient” therefore exists in Lake Vanda mats, with irradiance flux decreasing and nutrient concentration increasing inside the mat. The role of nutrients in controlling benthic microbial growth in Lake Vanda has not been addressed, though the plankton in the upper waters are considered strongly P-limited [16,18]. Elemental ratios of N:P reported here for microbial mats are high relative to the median of freshwater habitats, while C:N ratio is only slightly above median [42], supporting the view that P may limit mat growth. P-limited growth may constrain the rate of biomass accrual in the recently flooded parts of the lake, and the availability of recycled nutrients within mats may support internal metabolism.

The picture that emerges from our data, consistent across depths and between sampling occasions, is that benthic biomass begins to accrue as discreet annual laminae within a few years of the emergence of benthic substrate under ice. New laminae are derived by the upwards growth of intertwined bundles of *Leptolyngbya* trichomes, rich in carotenoids (particularly myxoxanthophyll), giving rise to a thin new orange-brown surface layer, which, during the late summer and winter, receives a variable dose of fine sedimentary material. These tufts occasionally form pinnacles, initially a mm or less tall, which slowly extend over time. Slow, undisturbed accumulation of biomass, layer by layer, perhaps limited by rate of nutrient accrual, provides a simple explanation of the patterns of benthic biomass by depth (and hence by time) in the upper waters of Lake Vanda. It appears that this process may be a quantitatively significant sink for biologically active elements in that lake.

The pattern of accumulation in the part of the lake where inundation history is known is consistent with that history. The multi-variable discontinuity at 14–16 m in the lake (in 2010) relates to a zone that has been submerged by a surge in lake level rise since 1970–1980. The mat characteristics at this discontinuity, thickness of 25 mm accumulating at 0.3 mm y^−1^ and pinnacle height of 10 mm accumulating at 0.2 mm y^−1^, are consistent with an age of ~50 years. At depths below the discontinuity, mats are substantially thicker with much larger pinnacles and a markedly higher organic carbon and carbonate content. Estimating the age of these mats requires extrapolation of relationships such as those in Figure 4. Thickness increase rates of 0.3 mm y^−1^ and pinnacle net extension of 0.2 mm y^−1^ suggest ages for 22 m mats of 60–100 years, though the questionable assumption that rates of change remain constant for such lengths of time renders estimates imprecise. 

Rising levels in the MDV lakes are best related to summer degree days above freezing [8]. In recent decades, the region appears to be undergoing a period when summer climate is sufficiently warm for lake level to have been steadily rising. Microbial mats confirm that this increase has been ongoing since the mid 20^th^ century and suggest that it may have begun perhaps 100 years ago. At the current rate of increase in lake level, microbial mats in Lake Vanda are able to keep pace with level rise in terms of initiating colonization. However, their slow rate of development means that they will take decades to reach their climax biomass and complexity. What is not addressed here is the fate of microbial mats in deeper parts of the lake where level change is affecting irradiance regime. There, a decline in microbial production can be expected as irradiance becomes increasingly limiting to sustainable biomass. Coring of the microbial mat at the junction between the two convection cells and below will allow the history of mat development to be better constrained, and sampling of mats from significantly below the chemocline will determine qualitative differences between the responses of mat communities there and those in the recently evolved upper cell.

## 5. Concluding Remarks

The level of Lake Vanda has been gradually rising for several decades, resulting in new habitat becoming available for colonization by microbial mats. This natural experiment allows temporal aspects of the development of these mats to be examined. Here, we show how mats are net sinks for nutrients and carbon in the lake, accumulating slowly through the elaboration of annual growth increments. Annual laminae retain photosynthetic capacity for many years after they have formed, acclimating to changing conditions associated with gradual burial under new layers. As mats grow, macroscopic structures emerge over decadal timescales from prostrate communities in the form of cm-scale, laminated pinnacles; at present, these are poorly developed in the upper 20 m of the lake compared to deeper waters. Rate of mat growth is such that, at the time of this study, areal biomass was not in equilibrium with ambient conditions; rather, the history of lake level change played a significant role in determining extant mat characteristics. The pace of change in Antarctica in relation to the rate at which ecosystems can respond will play a significant role in determining how ecosystems respond to climatic change.

## Supplementary Material

**Table S1:** Results of regression model fitting to time series data. Three functions were fitted to each variable, linear (Chla = aT), rectangular hyperbolic (Chla = a(T)/(b+T)); and sigmoid (Chl-a = a/(1+e^(−(T−b)/c)^), where T is time under perennial ice cover. In each case curves were fitted with and without intercepts. Cells indicate the adjusted r^2^ of the model fit and the *p*-value for the regression ANOVA in parentheses. In all cases, n = 6. Models were fitted using SigmaPlot 10.0 (Systat Software). The best fit for each variable is indicated in italics.

Model	Mean Chlorophyll-a	Mean Thickness	Median Pinnacle Height
Linear + intercept	0.90 (0.002)	0.96 (0.0003)	0.99 (<0.0001)
Linear - intercept	0.73 (>0.05)	0.95 (0.005)	0.95 (<0.0001)
Hyperbola +	0.92 (0.011)	0.95 (0.005)	0.99 (0.0003)
Hyperbola -	*0.93 (0.001)*	0.94 (0.001)	*1.00 (<0.0001)*
Sigmoid +	0.82 (>0.05)	0.99 (0.006)	1.00 (0.002)
Sigmoid -	0.92 (0.01)	*0.99 (0.0003)*	*1.00 (<0.0001)*

## Commentary

Interpretation of model fits is complicated, as accompanying time is a gradual deepening of the water and, thus, a change in growth conditions. In all three variables, rates of increase declined after several decades of growth, and whether this is due to deteriorating growth conditions (perhaps less irradiance as depth increased), limitation by self-shading or a combination of the two, it cannot be distinguished from our data.

Chlorophyll-a showed the best fit to the rectangular hyperbolic curves, with curve parameters suggesting that saturation would be achieved at 12.4 ± 3.2 µg cm^−2^, with half saturation biomass reached after 33 ± 14 years. That translates to an initial (years 0–33) rate of accrual of approximately 0.18 µg cm^−2^ y^−1^, slightly faster than that indicated by the linear + intercept model of 0.14 µg cm^−2^ y^−1^. A saturating hyperbolic model is consistent with rapid initial accumulation of chlorophyll-a, followed by a declining rate of accrual as biomass begins self-shading or as ambient irradiance declined.

Thickness was well described by all models, though the best fit was to a Sigmoid (logistic) function. Parameters suggest that inflection time from exponential early growth was at 25 years, when the mat would be 8 mm thick, with maximum thickness of 16 mm after ~50 years. Saturation of thickness would require annual growth to be balanced by annual loss, and this may not be reasonable for the Lake Vanda situation. Indeed, material thicker than 16 mm was found in the lake, and biomass appears to accumulate for much longer than 50 years. The accumulation rate of new mats may thus be governed by different processes than for older mature mats.

Pinnacle height was also well described by all models. The slightly better fit of the hyperbolic and sigmoid functions suggests that some degree of saturation of pinnacle size was developing as mats aged and as irradiance declined, as was suggested for thickness and chlorophyll-a. Hyperbolic and sigmoid models suggested that saturation would occur after 60 and 40 years, respectively, at a maximum median heights of approximately 20 mm. However, pinnacles of much taller than 20 mm were seen in the lake (Figure 3) and, as with thickness, pinnacle elongation during the development of new mats appears to be governed by other processes than those controlling development in older, mature mats.

**Table S2:** Microscopic characterization of cyanobacterial morphotypes in flat microbial mats at 10 and 18 m in Lake Vanda. +: rare (present in some fields); ++: frequent (present in all fields); +++: common (many trichomes in all fields)

			10 m			18 m		
Morphotypes	Description	Cell width(µm)	Brown	Green	Purple	Brown	Green	Purple
*Oscillatoria* cf *. sancta*		~ 8	+	+		+	+	+
*Phormidium* cf. *autumnale*		6–7	+	+	+	+	+	+
*Phormidium* cf. *murrayi*		4–5	+			+		
*Leptolyngbya* sp. 1	pointy end cell, longer than wide	0.5–1	+++	+++	+++	+	+	+
*Leptolyngbya* sp. 2	square cells, longer than wide	1–2	+++	+++	+++	+		
*Leptolygnbya* sp. 3	square cells, longer than wide, no constriction at cross-wall	1–2	+++	+++	++	+++	+++	+++
*Leptolyngbya* sp. 4	rounded end cell, longer than wide, constriction at cross-wall	1–2		++		+	+	
*Pseudanabaena* sp.		~2		+	+	+		+
Unicellular cyanobacterium cf. *Aphanocapsa*						+		
